# Bacteremia Caused by* Kocuria kristinae* from Egypt: Are There More? A Case Report and Review of the Literature

**DOI:** 10.1155/2016/6318064

**Published:** 2016-10-31

**Authors:** Reem M. Hassan, Dina M. Bassiouny, Yomna Matar

**Affiliations:** ^1^Department of Clinical and Chemical Pathology, Faculty of Medicine, Cairo University, Cairo, Egypt; ^2^Department of Psychiatry, Faculty of Medicine, Cairo University, Cairo, Egypt

## Abstract

*Kocuria kristinae* is opportunistic Gram-positive cocci from the family Micrococcaceae. It is usually considered part of the normal flora that rarely is isolated from clinical specimens. Here, we report a case of* Kocuria kristinae* bacteremia; to the best of our knowledge, this is the first report from Egypt.

## 1. Introduction

*Kocuria* are Gram-positive, coccoid actinobacteria that occur in tetrads belonging to the family Micrococcaceae, suborder Micrococcineae, order Actinomycetales [1]. They are widely distributed in nature and can also be found frequently as normal skin and oral cavity flora in humans and other mammals. The genus contains 18 species, only five of which are known to be opportunistic pathogens [2].

Few reports on* Kocuria* spp. clinical infections exist in the literature. They were reported to cause catheter-related bacteremia in immunocompromised patients and those with chronic illness, peritonitis, cholecystitis, and urinary tract infection in patients with indwelling urinary catheters [3–6].

The underestimated prevalence of this organism is due to its misidentification as coagulase-negative staph and absence of guidelines for its clinical evaluation as a pathogen as it can be a common source of contamination in clinical specimens [7, 8].

## 2. Case Report

A 70-year-old female patient diagnosed with bipolar disease was admitted to the psychiatry department in Cairo University Hospital (Kasr Al-Ainy). The patient develops manic/depressive episodes every now and then and was in an episode of mania for which she received treatment for 2 weeks, regular antipsychotic regimen (risperidone, depakine, and quetiapine). A peripheral cannula was introduced for intravascular fluids. The patient was not controlled with regular treatment and received aqueous intramuscular injection of clopixol 200 mg, after which she developed bilateral lower limb weakness, disturbed conscious level a day later, and fever. Weakness progressed to hypotonia and external rotation, with no rigidity and equivocal plantar reflex. Manifestations in the right limb were severer than in the left one, as there were redness, warmness, and edema of calf muscles but no pain or tenderness. The patient received clexane 40 mg prophylaxis. Examination of the patient revealed temperature 38.3°C, blood pressure 130/80 mmHg, pulse 110/min, and respiratory rate 20/min.

Radiological investigations were done in the form of CT brain, MRI brain, and echocardiogram, which were normal, and venous Duplex on lower limbs that showed recent adherent deep venous thrombosis (DVT) in right peroneal and soleal areas for which she received a higher dose of clexane (60 mg every 12 hours).

Laboratory investigations were done and results were as follows: CPK was 3200 U/L which gradually decreased later on, AST was 94 U/L, platelets were 87000 cells/cmm, Na was 134 mg/dL, K was 3.5 mg/dL, Ca++ was 0.61 mg/dL, Hb was 10 mg/dL, WBCs were 3600 cells/cmm, CRP was positive, PT and PTT were normal, and urine culture grew* E. coli* (ESBL).

**Figure 1:**
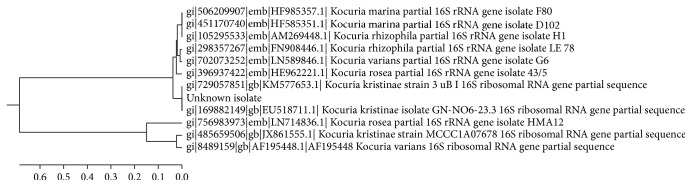
Evolutionary relationships of taxa. The evolutionary history was inferred using the UPGMA method [10].

*Microbiological Methods.* Blood culture was withdrawn in the BACTEC Plus aerobic/F and BACTEC Plus anaerobic/F blood culture bottles (Becton, Dickinson and Company, Spain, MD). All bottles were incubated in BACTEC 120 instrument. Blood culture grew Gram-positive cocci on blood and chocolate agar with catalase-positive, coagulase-negative, and typical pigmentation of* Kocuria* colonies (pale rose) that became more distinct after further 24 hr incubation in 4°C. Vitek2 automated identification system (bioMerieux, France) was used to identify the isolate using the Gram positive identification cards (GP cards) and the isolate was identified as* Kocuria kristinae* (99%). Susceptibility testing was done using the modified Kirby-Bauer disc diffusion method; the organism was found to be sensitive to cefoxitin, gentamicin, amikacin, ciprofloxacin, levofloxacin, and linezolid but resistant to vancomycin, teicoplanin, rifampicin, amoxicillin/clavulanate, and clindamycin. Phenotypic identification was confirmed by performing a molecular assay, namely, 16S rRNA gene sequencing, as previously described using the primer sets 536f 5′CAGCAGCCGCGGTAATAC and RP2 5′eACGGCACCTTGTTACGACTT (AccuOligo, Bioneer, Daejeon, Korea), BigDye® Terminator v3.1 cycle sequencing kit (Applied Biosystems, Foster City, CA, USA), and the BigDye Xterminator™ purification kit (Applied Biosystems, Foster City, CA, USA), and then run on Applied Biosystems 3500 Genetic Analyzer (Applied Biosystems, Foster City, CA, USA). Sequences were analyzed with AutoAssembler software (KB_3500_POP7_BDTv3.mob) and compared using the basic local alignment search tool. Also, a neighbor-joining phylogenetic tree with the 16S rRNA gene sequences of all* Kocuria* species using MEGA6 program (Figure 1) was constructed [9–12].

## 3. Discussion

By reviewing the literature, 20 reports on* Kocuria* infections in humans were found, most of which were in immunocompromised hosts with few reports in otherwise healthy people. The five opportunistic* Kocuria* spp. are* K. kristinae*,* K. rhizophila*,* K. rosea*,* K. varians*, and* K. marina* [3, 13–16].

*K. kristinae* was first described in 1974 (previously known as* Micrococcus kristinae*). The bacterium is facultative anaerobic, nonmotile, catalase-positive, and coagulase-negative and is known to cause catheter-related bacteremia and infective endocarditis [2, 17].

In the present case report, we describe a case of catheter-related blood stream infection caused by* K. kristinae*, complicated by DVT, which was the case in other reported infections caused by* Kocuria* spp. As the patient had a concomitant urinary tract infection (caused by* E. coli*), the presented fever could be caused either by one of the organisms or even by the DVT. The patient was not known to be immunocompromised except for antipsychotic drugs which with prolonged use can cause impaired liver functions with mild state of immune system disturbance.

Infection with this organism takes place mostly in immunocompromised patients like* K. kristinae* bacteremia that occurred in a patient suffering from ovarian cancer. This patient had multiple febrile episodes through a period of six months, all of which grew the organism from several blood cultures and central venous lines (CVLs) [18].

*K. kristinae* was also described as the cause of acute peritonitis in a patient with end-stage renal failure that had CAPD for two years. The source of contamination was suspected to be touch of the catheter that led to access of bacteria into the peritoneal cavity [4, 19].

An immunocompetent pregnant female developed severe* K. kristinae* intravascular infections with suppurative thrombosis that led to septic pulmonary emboli. These complications followed catheter-related blood stream infection [3].

A recent report of* K. kristinae* bacteremia discussed an infant with a history of prolonged diarrhea complicated with black hairy tongue symptoms [20].

A different access route of* K. kristinae* infection was recently documented in an elderly diabetic patient who developed endocarditis after amputation of a forefoot ulcer and the central venous catheter was not involved [21].

Many recent studies, including ours, correctly identified* Kocuria* spp. using the Vitek-2 ID-GPC Gram-positive identification card, perhaps due to the recently introduced larger database that allows the identification of additional taxa [22].

Misidentification among members of the* Kocuria* genus cannot be ruled out as other studies have reported such situations [5, 13, 14].

As a consequence to the absence of evidence-based guidelines for managing* Kocuria* infections, cases are managed depending on previous experience or similar cases in the literature. These reports suggested the removal of offending catheter and the use of an antibiotic either alone or in combination. Only one report by Szczerba proposed amoxicillin/clavulanate along with drugs like ceftriaxone, cefuroxime, doxycycline, and amikacin as a first-line therapy against micrococcal infections [23].

Also, there are no specific criteria for interpreting sensitivity assays with* Kocuria* isolates and only a few investigated the resistance mechanisms expressed in this genus as the one report that postulated decreased cell wall permeability and multidrug efflux pump expressed in these organisms [2]. Another study identified proteins that may be involved in efflux mechanisms [24].

To summarize,* Kocuria kristinae* bacteremia should be considered especially in liable patients and on repeated isolation. Introduction of newer diagnostic techniques to the microbiology lab leads to better identification of rare pathogens and underestimated ones. Proper diagnosis is the key to better treatment strategies.
